# Exogenous Sequences in Tumors and Immune Cells (Exotic): A Tool for Estimating the Microbe Abundances in Tumor RNA-seq Data

**DOI:** 10.1158/2767-9764.CRC-22-0435

**Published:** 2023-11-21

**Authors:** Rebecca Hoyd, Caroline E. Wheeler, YunZhou Liu, Malvenderjit S. Jagjit Singh, Mitchell Muniak, Ning Jin, Nicholas C. Denko, David P. Carbone, Xiaokui Mo, Daniel J. Spakowicz

**Affiliations:** 1 Division of Medical Oncology, The Ohio State University Comprehensive Cancer Center, Columbus, Ohio.; 2 The Pelotonia Institute for Immuno-Oncology, The Ohio State University Comprehensive Cancer Center – James Cancer Hospital, and Solove Research Institute, Columbus, Ohio.; 3 Department of Biomedical Informatics, The Ohio State University College of Medicine, Columbus, Ohio.

## Abstract

The microbiome affects cancer, from carcinogenesis to response to treatments. New evidence suggests that microbes are also present in many tumors, though the scope of how they affect tumor biology and clinical outcomes is in its early stages. A broad survey of tumor microbiome samples across several independent datasets is needed to identify robust correlations for follow-up testing. We created a tool called {exotic} for “exogenous sequences in tumors and immune cells” to carefully identify the tumor microbiome within RNA sequencing (RNA-seq) datasets. We applied it to samples collected through the Oncology Research Information Exchange Network (ORIEN) and The Cancer Genome Atlas. We showed how the processing removes contaminants and batch effects to yield microbe abundances consistent with non–high-throughput sequencing–based approaches and DNA-amplicon–based measurements of a subset of the same tumors. We sought to establish clinical relevance by correlating the microbe abundances with various clinical and tumor measurements, such as age and tumor hypoxia. This process leveraged the two datasets and raised up only the concordant (significant and in the same direction) associations. We observed associations with survival and clinical variables that are cancer specific and relatively few associations with immune composition. Finally, we explored potential mechanisms by which microbes and tumors may interact using a network-based approach. *Alistipes*, a common gut commensal, showed the highest network degree centrality and was associated with genes related to metabolism and inflammation. The {exotic} tool can support the discovery of microbes in tumors in a way that leverages the many existing and growing RNA-seq datasets.

**Significance:**

The intrinsic tumor microbiome holds great potential for its ability to predict various aspects of cancer biology and as a target for rational manipulation. Here, we describe a tool to quantify microbes from within tumor RNA-seq and apply it to two independent datasets. We show new associations with clinical variables that justify biomarker uses and more experimentation into the mechanisms by which tumor microbiomes affect cancer outcomes.

## Introduction

The microbiome affects many aspects of human health, including cancer. Interactions with the immune system affect immune cell activity and the levels of systemic inflammation, and thereby various disease states (1). For example, gut microbiome samples collected before treatment with immune checkpoint inhibitors could predict tumor response months later (2–4). Intrinsic tumor microbes have been established for years in a relatively small number of cancers. However, recent studies identified their presence in a broader set of histologies and the ability to use high-throughput sequencing data for their study. Understanding the directionality of causal relationships may yield new approaches to cancer treatment.

Causal connections between tumor microbes and cancer outcomes remain relatively few. For example, *Bacteroides fragilis* biofilms on colon polyps have been found to secrete a toxin that directly damages DNA and leads to colon cancer (5). In other cases, *Helicobacter pylori* secrete molecules that elicit an inflammatory cascade that has been shown to drive tumorigenesis in gastric adenocarcinoma (6). The fungal genus *Malassezia* was shown to drive pancreatic ductal adenocarcinoma growth by activating the C3 complement pathway (7). Broader surveys include a recent bacterial ribosome amplification-based approach applied to six types of tumors, which found many microbes (8). Hundreds of negative controls and paraffin-only blocks were sequenced to establish the background signal and system contamination. Furthermore, FISH and IHC validated the presence of microbes in many locations in tumor tissues and cells. This seminal work opened the door to studying the microbes’ provenance and effects on cancer outcomes, such as by leveraging high-throughput sequencing datasets.

A recent survey of cancer tissue whole-genome shotgun sequencing data from the publicly available data resource The Cancer Genome Atlas (TCGA) that used a similar approach to the {exotic} tool (described here) found bacteria and viruses in all tumors tested (9). Furthermore, these exogenous sequences were cancer specific, and exogenous sequences in blood could predict the type of cancer. Provocatively, this was even true in early-stage colon cancer samples, where no circulating tumor sequences were detectable in the blood samples. This powerful observation was lacking by (i) focusing on DNA-based observations, which may be more prone to contamination; (ii) excluding fungi, which have been shown to affect several cancers; (iii) not validating on an independent dataset; and (iv) not testing other correlations that might confound the association with tumor type, such as the microenvironment.

We extend this previous work with a computational tool we call {exotic} for exogenous sequences in tumors and immune cells. The process involves stringent alignment parameters and a series of filtering steps. We show how validation on two large, independent datasets—TCGA and Oncology Research Information Exchange Network (ORIEN)—finds microbes with concordant behavior in cancer types.

## Materials and Methods

### ORIEN Study Design and Sequencing Methods

ORIEN is an alliance of 18 U.S. cancer centers established in 2014. All ORIEN alliance members utilize a standard Total Cancer Care (TCC) protocol. As part of the TCC study, participants agree to have their clinical data followed over time, undergo germline and tumor sequencing, and are contacted by their provider if an appropriate clinical trial or other study becomes available (10). TCC is a prospective cohort study with a subset of patients enrolled in the ORIEN Avatar program, which includes research use only grade RNA sequencing (RNA-seq) and collection of deep longitudinal clinical data with lifetime follow-up. M2GEN, ORIEN's commercial and operational partner, harmonizes all abstracted clinical data elements and molecular sequencing files into a standardized, structured format to enable aggregation of deidentified data for sharing across the network. A total of 480 ORIEN Avatar patients diagnosed with melanoma, colorectal, genitourinary, renal cell carcinoma, sarcoma (SARC), and thoracic cancer were included in this study. ORIEN Avatar specimens underwent nucleic acid extraction and sequencing at HudsonAlpha or Fulgent Genetics. Qiagen RNAeasy plus mini kit was performed for frozen tissue, generating a 216 bp average insert size. For formalin-fixed paraffin-embedded (FFPE) tissue, a Covaris Ultrasonication FFPE DNA/RNA kit was used to extract DNA and RNA, generating a 165 bp average insert size. RNA was selected using the Illumina TruSeq RNA Exome with single library hybridization, followed by cDNA synthesis, library preparation, and sequencing (100 bp paired reads at Hudson Alpha, 150 bp paired reads at Fulgent) to a coverage of 50M paired reads. Adapters were trimmed via k-mer matching, followed by quality trimming and filtering, contaminant filtering, sequence masking, GC filtering, length filtering, and entropy filtering. Cleaned reads were aligned to the human genome reference (GRCh38/hg38) and the Gencode genome annotation v32 using STAR (11).

### Data Processing, Contaminant Removal, and Normalization

Bulk RNA-seq samples with corresponding expression data were obtained from ORIEN and TCGA. Samples from TCGA were identified and downloaded using the R package {GenomicDataCommons} and restricted to samples from patients’ primary tumors. TCGA projects included lung adenocarcinoma (LUAD), LUSC, colorectal (COAD), READ, SARC, THCA, skin (SKCM), kidney renal cell carcinoma (KIRC), and bladder (BLCA). These samples were then classified using Kraken2 with Bracken, and a custom database was built using the available libraries archaea, bacteria, fungi, human, plasmid, protozoa, UniVec, and viral. Microbes with greater than five supporting reads were included in the abundance tables. From here, the R package {decontam} was used to identify contaminants within ORIEN and TCGA samples (12). These contaminant lists were revised using the literature-reviewed list from Poore and colleagues before contaminants were removed from the count tables (9). The counts were then normalized using VOOM-SNM, treating cancer type as a biological variable to be preserved and the sequencing center (TCC = ORIEN, UNC = TCGA) and sample preservation method [FFPE vs. fresh frozen (FF)] as technical variables whose variance should be removed. The relative abundances of all human and microbe portions of the samples are then calculated. All prevalences are calculated from rarefied, decontaminated counts.

### 16S Sequencing and Analysis

The bacterial 16S rRNA gene was amplified from FF tumor (*n* = 31) and adjacent normal (*n* = 31) tissues from 31 patients. Tissues were lysed on a PowerLyzer 24 at 2,000 rpm for 30 seconds, and then DNA was purified using an AllPrep mini kit (QIAGEN). The bacterial rDNA was amplified using V3-V4 primers and KAPA HiFi enzyme (50°C 30 seconds, 72°C 2 × 20 cycles). Amplicons were cleaned by magnetic beads, and then sequencing libraries were generated using a QIAseq kit (QIAGEN) following the manufacturer's instructions. Libraries were sequenced on a MiSeq 2 × 300 (600 cycles) using a V3 reagent kit (Illumina). Demultiplexed fastqs were filtered for quality and length (340–440 bp). Taxonomy was assigned by processing through the precontamination filtering steps of {exotic} pipeline.

### Correlation/other Modeling Methods, Including Survival

All microbe relative abundance correlations with age, Buffa hypoxia score, body mass index (BMI), and all immune cell fractions were tested using Spearman ranked correlation test. Unnormalized expression counts were deconvolved to immune cell fractions using CIBERSORT (13). Survival analyses started from rarefied prevalence tables, whereby counts were subsampled to the lowest common read depth. In the case of the rarefied tables, any reads supporting the presence of an organism was grouped as “present.” Cox proportional hazards models for overall survival were stratified by microbe prevalences and the significance tested by log-likelihood. These tests were performed independently in TCGA and ORIEN datasets. The results were then compared to find microbes that were concordant in both datasets with respect to significance and the direction of effect between the groups, with false discovery rate correction by the method of Benjamini and Hochberg (14).

### Network Construction and Analysis

All microbes were correlated with all genes using Spearman ranked correlation test in both the TCGA and ORIEN datasets. The ORIEN results were filtered to keep only correlations that had the same direction of effect as the corresponding correlation in TCGA data. The top and bottom 2.5% correlations were then used to define the edges of a network as the most extreme correlations. The degree centrality of this network was calculated as the number of connections formed with each node, and the betweenness centrality was calculated using the R package igraph (RRID:SCR_021238). Microbes with high degree centrality and literature significance were chosen for pathway analysis with R package FGSEA (bioRxiv 060012) using the hallmark pathway database. The microbes were given indicators for each of the correlation analyses, in which they were found to be consistently significant in TCGA and ORIEN datasets. These indicators were then summarized in a table with the degree and betweenness centrality information from the network. The concordant links per node histogram was compared with a random network by creating two datasets each with two matrices of 100 × 100 and 100 × 1,000 to represent microbes and genes, respectively. These two matrix sets were processed by the same method as the tumor microbe and gene data, including correlation, concordance filter, most extreme 5% selection, and then degree centrality calculation.

### Data Availability Statement

The Ohio State University Institutional Review Board approved data access in an Honest Broker protocol (2015H0185) and Total Cancer Care protocol (2013H0199) in coordination with Aster Insights. The processed data generated in this study are publicly available in Gene Expression Omnibus through the BioProject PRJNA856973. The {exotic} tool is available at: https://github.com/spakowiczlab/exotic. Analysis scripts to regenerate all figures and tables are available at: https://github.com/spakowiczlab/exotic-manuscript.

## Results

### Description of the Dataset

We processed tumor RNA-seq from 480 samples through the {exotic} tool (Table 1). These included LUAD (*n* = 99), SARC (*n* = 116), COAD (*n* = 70), KIRC (*n* = 20), and others. We retrieved and processed by the same method the equivalent cancer types from TCGA. TCGA samples showed similar BMI at the time of collection and genders but were different in the age of collection (average age of ORIEN 60.13 vs. 64.9 in TCGA, *t* test *P*-value < 0.001). In addition, the fraction of samples preserved by freezing was nearly 100% of TCGA samples, whereas roughly one-quarter of the ORIEN samples were FFPE (*χ*^2^*P* value < 0.001). The fraction of samples from primary tumors was also significantly different between the cohorts, with the ORIEN cohort having a larger perfect from metastatic legions (primary 65.4% vs. 931% in ORIEN and TCGA, respectively). Finally, the times to last follow-up or death were significantly different, with the ORIEN cohort showing nearly two years longer (*t*-test *P* value <0.001).

**TABLE 1:** Data summary

	ORIEN	TCGA	*P*
*N*	480	2,993	
BLCA (%)	19 (4.0)	414 (13.8)	
COAD (%)	70 (14.6)	478 (16.0)	
KIRC (%)	20 (4.2)	538 (18.0)	
LUAD (%)	99 (20.7)	533 (17.8)	
LUSC (%)	3 (0.6)	502 (16.8)	
READ (%)	20 (4.2)	166 (5.5)	
SARC (%)	116 (24.3)	259 (8.7)	
SKCM (%)	16 (3.3)	103 (3.4)	
OTHER (%)	117 (24.4)	0 (0.0)	
AGE AT COLLECTION [MEAN (SD)]	60.13 (12.60)	64.93 (11.83)	<0.001
GENDER = MALE (%)	275 (57.3)	1,796 (60.1)	0.271
BMI AT COLLECTION [MEAN (SD)]	28.67 (6.56)	28.43 (13.44)	0.728
PRESERVATION = FROZEN (%)	376 (78.3)	2,962 (99.0)	<0.001
OVERALL SURVIVAL (DAYS) [MEAN (SD)]	1,569.70 (1074.73)	762.70 (726.24)	<0.001
TISSUE SOURCE = PRIMARY (%)	314 (65.4)	2785 (93.1)	<0.001
TUMOR STAGE (%)			<0.001
Stage 1	77 (16.1)	921 (40.4)	
Stage 2	98 (20.5)	647 (28.4)	
Stage 3	125 (26.2)	501 (22.0)	
Stage 4	112(23.5)	208 (9.1)	

### Description and Validation of the {exotic} Tool

**FIGURE 1:**
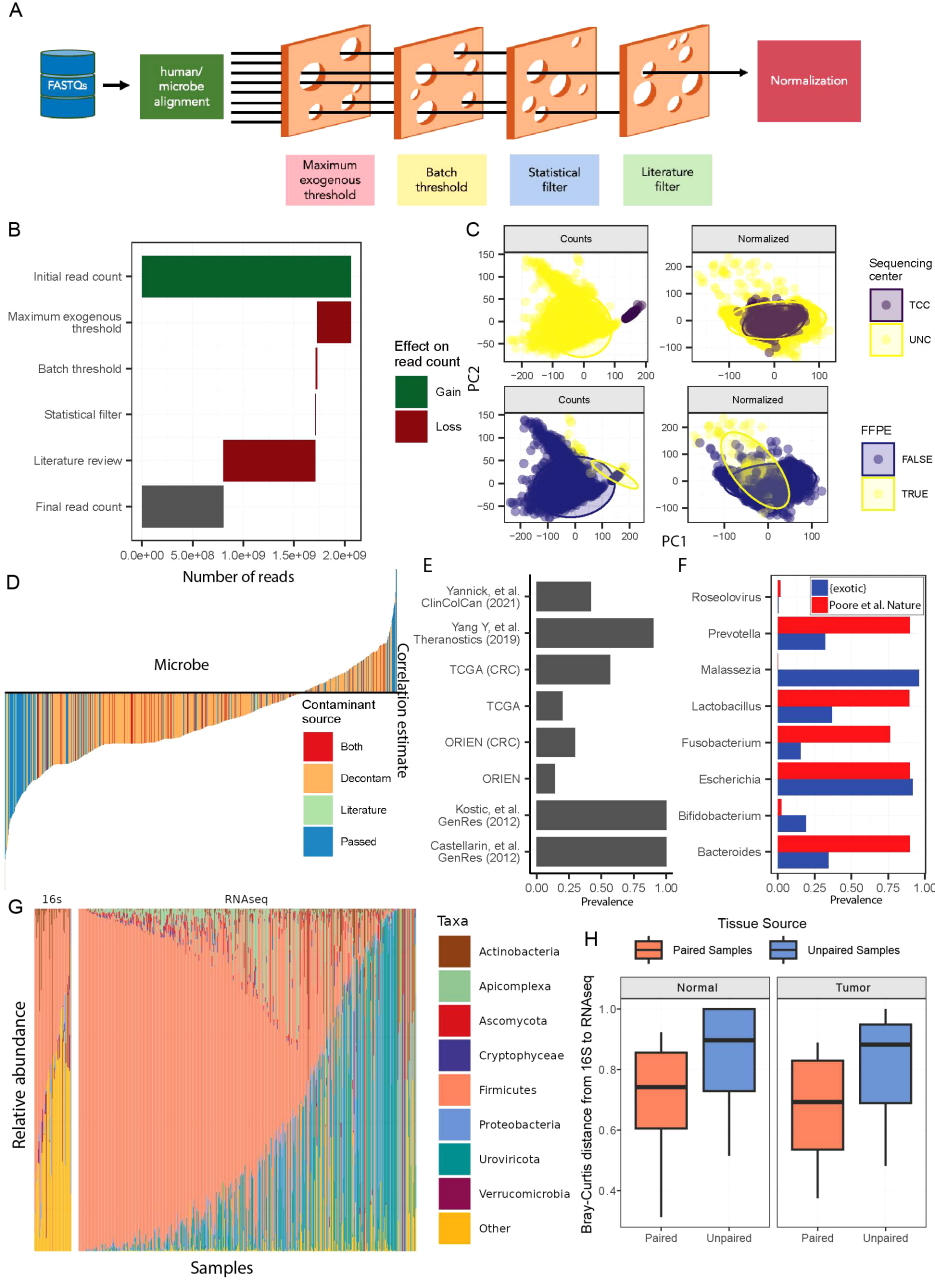
Summary and validation of the {exotic} tool. **A,** Schematic of the {exotic} tool, showing the process of aligning raw RNA-seq FASTQs to databases for human and microbial identification, followed by filtering to remove contaminants. Filtering steps include the removal of samples with a high percentage of exogenous reads and samples from small batches that prevent contaminant checks, as well as reads that align to microbes found to be contaminants by statistical or literature review–based filtering. The remaining samples’ microbe counts are normalized by VOOM-SNM. **B,** The loss of reads at each step of the {exotic} tool. **C,** Normalization removes batch effects from the sequencing center (all ORIEN samples were sequenced at TCC, and all TCGA at UNC) and preservation method from decontaminated counts. **D,** Microbe-level filtering removes taxa that align with input RNA concentration and are consistently found in negative controls. Microbes with strong literature precedence as commensals are returned. **E,** Comparison of the prevalence of *Fusobacterium* found by {exotic} compared with literature values captured by non–RNA-seq–based approaches, with a much higher prevalence appearing in colorectal samples compared with all cancer types. **F,** A comparison of the prevalence of various taxa to those reported by Poore and colleagues (9) for TCGA dataset finds mostly lower prevalences of bacteria and viruses but includes the identification of fungi (*Malassezia*). **G,** The abundance of microbes found in a 16S-based validation dataset compared with the RNA-seq–based approach. **H,** Comparison of the distances between microbes identified by 16S and RNA-seq for samples from the same patient and tumor (Paired = TRUE, Tumor) and adjacent normal tissue (Paired = TRUE, Normal) versus samples from different patients (Paired = FALSE).

We designed the {*exotic}* tool to broadly but conservatively identify microbes present in the tumors and remove as many technical artifacts and contaminants from the dataset as possible (Fig. 1). The tool first maps raw reads with quality scores (FASTQ) to the human reference genome, with a second alignment pass following the standard workflow of TCGA and other large-scale sequencing efforts (Fig. 1A). Next, {exotic} aligns the unmapped reads to a wide range of non-human genomes, including bacteria, archaea, viruses, fungi, and a subset of other eukaryotes. Next, {exotic} filters contaminants in four phases that include two threshold-based sample filters and two microbe-based filters. First, samples with a total microbial load larger than 20% of the human reads are discarded as extreme outliers. Second, the remaining samples are categorized into batches according to sequencing site, preservation method, and flow cell, and batches with fewer than 10 samples are removed. Third, {exotic} removes taxa whose species-level relative abundances significantly correlate with input RNA concentration (12). Furthermore, {exotic} removes a list of organisms commonly found in sequenced negative controls (15), sparing taxa with strong literature precedence for host interactions (9). Finally, the outputs are normalized to remove technical artifacts. The filters are modular, enabling users to select those most appropriate to their dataset. For example, analyses with sequenced negative controls from a uniformly collected dataset may eschew the “Literature filter,” which both removes a large fraction of microbes and relies on data generated at other sites. Most analyses rely on intersecting results across multiple datasets in a discovery and validation-type approach. Here we rely on data from the ORIEN, which has the benefit of robust clinical data to support the analyses (Table 1).

The {exotic} tool discards roughly half of the non-human reads in processing (Fig. 1B). Roughly 10% of the total reads in the starting dataset were lost when filtering samples with a large fraction of microbes. A relatively small fraction of the reads is lost in the statistical filtering step, though this is a large number of microbial species (Fig. 1B and D). In contrast, literature-based filtering removes a large fraction of the reads but relatively few taxa (Fig. 1B and D). After processing and normalization, technical artifacts related to the sequencing center and sample fixation method (FFPE, FF) are removed (Fig. 1C).

The cleaned dataset identified microbes in quantities consistent with previous reports using other methods. For example, the fraction of samples containing *Fusobacterium* in both the ORIEN and TCGA colorectal [COAD, READ (colorectal cancer)] datasets were consistent with published reports using quantitative PCR-based measurements (16, 17) but lower than reports from amplicon sequencing (18), or microscopy (ref. 19; Fig. 1E). The amount of *Fusobacterium* found in colorectal cancer tumors was greater than in non-colorectal cancer tumors. However, the {exotic} tool found a lower prevalence of *Fusobacterium* than has recently been reported by DNA-based tumor microbiome analyses of TCGA data by Poore and colleagues (Fig. 1F; ref. 9). The {exotic} tool found similar prevalences of various common bacteria and viruses. Notably, {exotic} found several fungi to be highly prevalent, including *Malassezia,* which had been excluded in some previous efforts in this space (Fig. 1F; refs. 9, 20), though more recent efforts corrected this (20). The cleaned output included over 1,000 unique species across both datasets, including bacteria, fungi, viruses, and archaea. More species were observed in the FF samples than in FFPE (757 vs. 647).

To further assess the accuracy of the RNA-seq–based approach, we retrieved a subset of the tumors and adjacent normal tissue (*n* = 31 of each) and sequenced them by 16S DNA amplicon sequencing. Broadly, the fraction of reads belonging to major bacterial phyla, such as Firmicutes, was largely consistent between the two data types (Fig. 1G). Notable differences include a large number of bacteriophages (Uroviricota) found in the RNA-seq, which cannot be observed by 16S-based approaches. The 16S-based microbes were most similar to RNA-seq data from the same patient and tumor, followed by data from adjacent normal tissue from the same patient (Fig. 1H, “Paired”) than from different patients (“Unpaired”).

### Association of the Tumor Microbiome with Survival and Clinical Features

**FIGURE 2:**
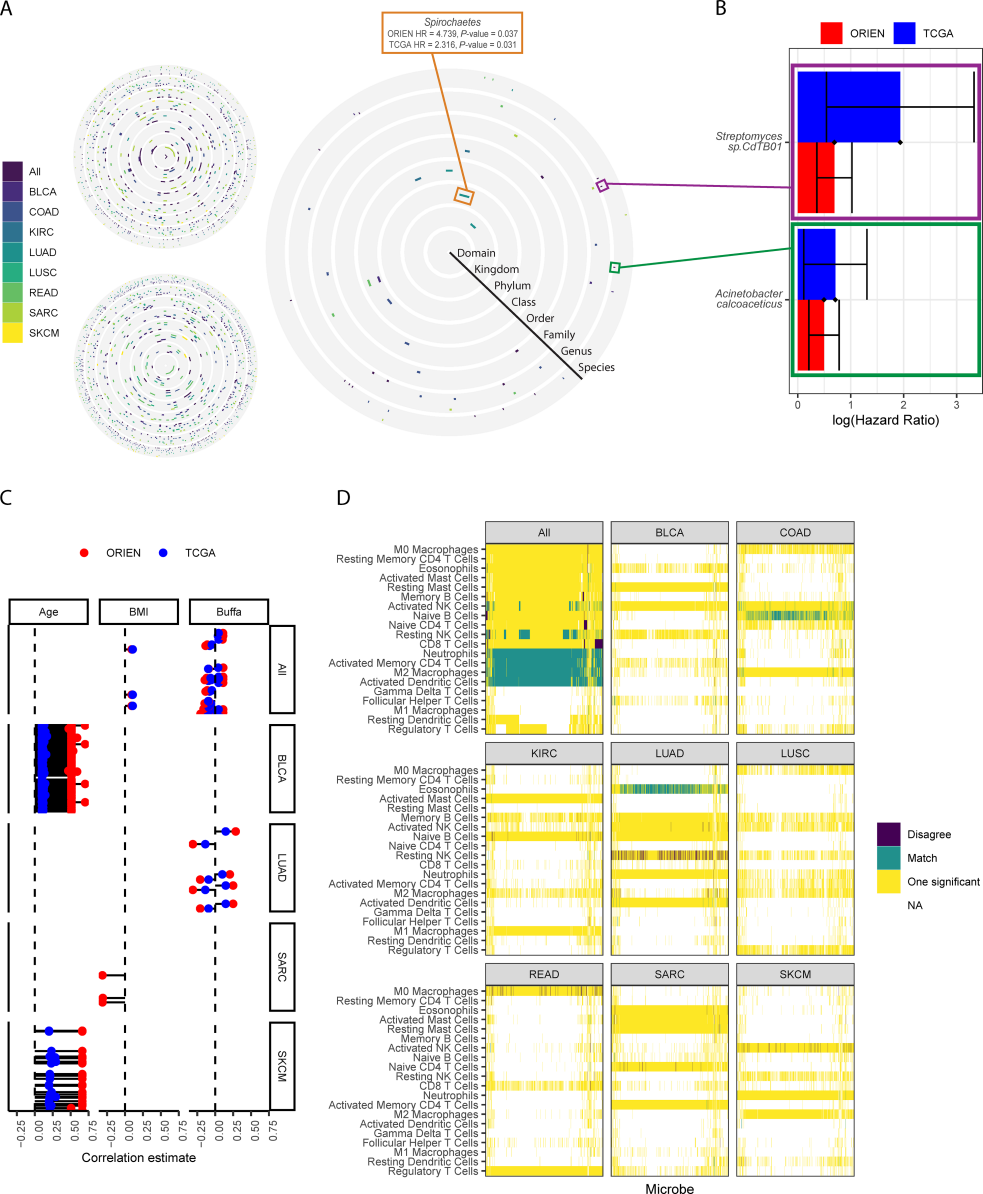
Estimating the clinical relevance of observed tumor microbes through the concordance between two independent datasets. **A,** Survival analyses by cancer stratified by microbe presence/absence at every taxonomic level. Colors indicate a significant association with survival. The ORIEN and TCGA analyses separately show many significant associations, whereas few are consistent (significant and affecting survival in the same direction) across both datasets. **B,** Examples of two taxa that show concordant associations between the two datasets. **C,** Microbes correlate with representative clinical variables and an expression signature of the tumor microenvironment (Buffa hypoxia score). Shown are concordant correlations for ORIEN and TCGA data, except for BMI, which is missing in TCGA for many cancers. **D,** Correlation of microbes with deconvolved immune cell abundances within each cancer.

Having identified, filtered, and normalized the microbial abundances, we sought to assess their relevance to human health by exploring associations with clinical features and outcomes. First, we target the association of microbes with overall survival in each cancer individually and an “all” group that includes every sample. Stratifying samples by the presence of each microbe at every taxonomic level revealed hundreds of significant associations across the ORIEN and TCGA datasets (Fig. 2A). The results are displayed as circles, where each ring refers to a taxonomic level (e.g., Kingdom, Phylum, etc.), and a colored bar indicates a significant association with survival, colored by cancer type. Both ORIEN and TCGA showed many associations with survival (many colored bars). Following a discovery and validation approach, we intersected the results to identify concordant associations by significance and whether there was a positive or negative effect on survival. Few microbes are associated with overall survival, and very few across more than one cancer type, suggesting cancer specificity or insufficient sample size (Fig. 2A; Supplementary Table S1). In cases where a microbe was associated with the “all” group, it was most often associated with one other cancer, which likely drove the effect in the context of the larger dataset. For example, the *Streptomyces* strain CdTB01 is associated with lower overall survival in KIRC, LUAD, and lung tumors across the entire dataset (Fig. 2B). This *Streptomyces* strain was later found to have a high betweenness centrality in a network constructed from microbe gene correlations. *Acinetobacter calcoaceticus* presence was also associated with reduced overall survival across all tumors, with a similar HR between the ORIEN and TCGA datasets. We further assessed a subset of these prevalence-based concordant results using their abundances and a multi-threshold survival approach (21). The taxa are significantly associated with survival at a range of abundances (Supplementary Fig. S1).

Next, we looked for general trends with clinical variables using a correlation-based approach. The relative abundance of microbes correlated with some, but not all, patient and tumor characteristics. Moreover, we observed enrichment of correlations in certain cancers instead of even distribution across the dataset (Fig. 2C). Again, this analysis relied on concordant observations, requiring that correlation was (i) statistically significant and (ii) in the same direction. Age positively correlated with many microbes in the BLCA and SKCM cancers but no others. The significant correlations were positive, with an increased relative abundance of a microbe associated with increased age. In contrast, BMI correlations were positive and negative (Fig. 2C). However, similar cancer specificity was observed, with significant correlations restricted to BLCA, LUAD, and SARC cancer types. Only in the case of BMI, did we observe significant correlations for ORIEN-only, as TCGA dataset is missing many values. Finally, we show an association with an expression signature of hypoxia (22). Again, we observed cancer specificity, with bidirectional correlations in LUAD and the entire dataset and only negative correlations in KIRC (Fig. 2C). Finally, we explored relationships between microbes and deconvolved immune cell abundances. No immune cells significantly correlated with microbes in both datasets and directionally agreed across all cancer types. Neutrophils, activated CD4 memory T cells, M2-type macrophages, and activated dendritic cells significantly correlated and agreed across the entire dataset (Fig. 2D). However, only naïve B cells and eosinophils significantly correlated and agreed in COAD and LUAD, respectively. Microbes are significantly associated with immune cells in opposite directions in the case of resting natural killer cells in LUAD and M0 macrophages in READ. More correlations agreed between the two datasets than disagreed, particularly when surveyed across the entire dataset.

### Tumor Microbiome–host Interactions Through Gene-microbe Networks

**FIGURE 3:**
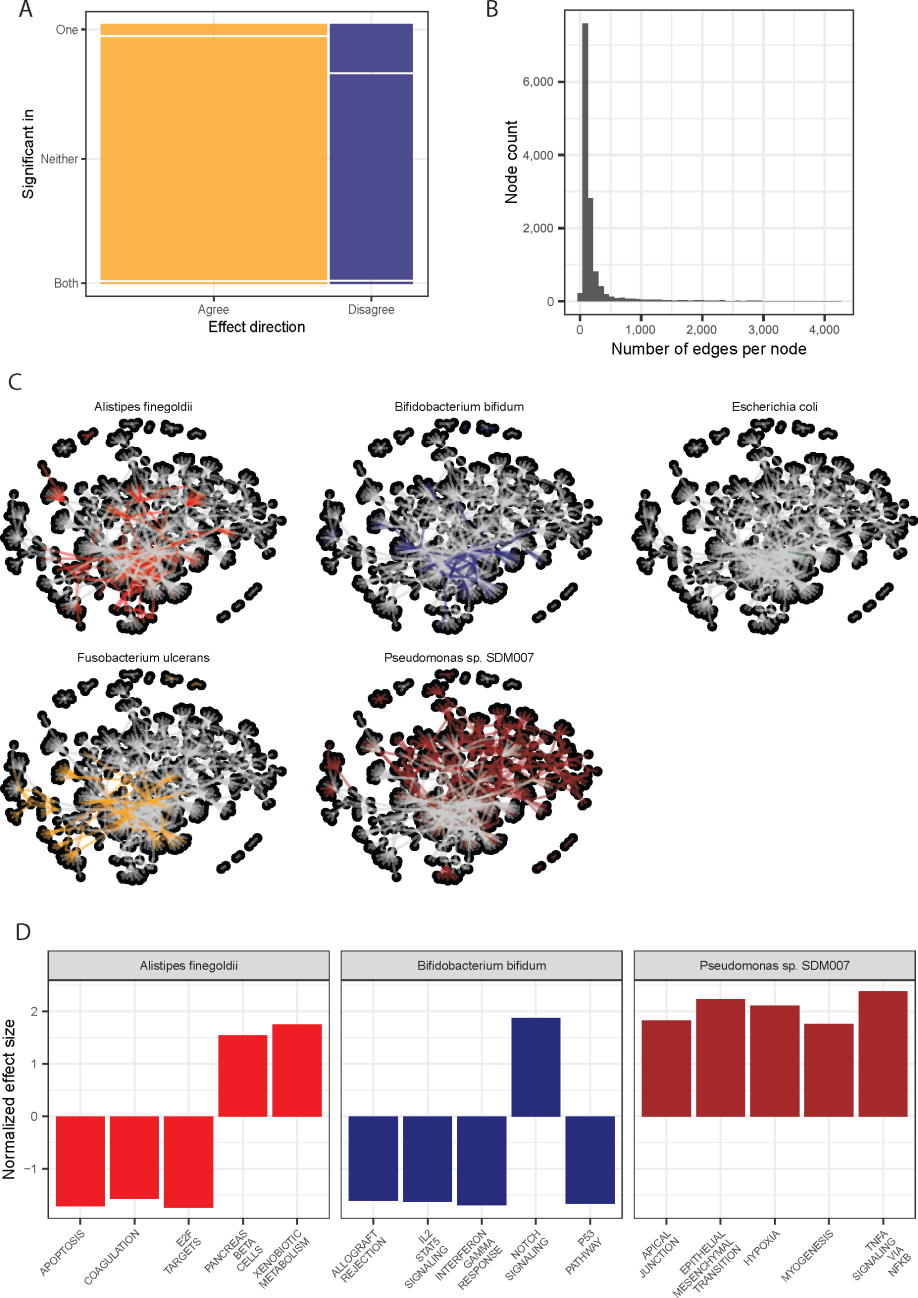
Network-based exploration of microbe–gene interactions. **A,** Summary of the correlations between all microbes and all genes, selecting only the correlations significant in both datasets and where the effect direction agrees. **B,** Consistent correlations are further filtered for the 5% most extreme values and then used to build a network for which the number of edges per node follows a power-law distribution. **C,** The top taxa by degree centrality are shown, which include those with precedence for affecting cancer outcomes (*Bifidobacterium*), as well as microbes that have only been described in the gut (*Alistipes*). Each microbe shows a distinct pattern of gene relationships (colored lines), suggesting a variety of interaction types. **D,** Examples of pathway enrichment analyses of the genes interacting with microbes include *Alistipes finegoldii, Bifidobacterium bifidum,* and a strain of *Pseudomonas.*

To explore the mechanisms by which microbes may associate with clinical outcomes such as survival and hypoxia, we related microbe abundances with gene expression, controlling for cancer type. We used a network-based approach to further reduce the FDR from pairwise associations (23). We correlated all microbes and all genes across all samples separately in each dataset. The correlation direction agreed in nearly 75% of the cases, with a small fraction significant in both (Fig. 3A). We then constructed a network drawing edges by the most extreme, significant, and concordant correlations (top 5% of concordant correlations; Supplementary Fig. S2). The number of edges per node followed a power-law distribution (Fig. 3B). To illustrate the difference between this power-law distributed network and a random network, we created a random network following the same concordance rules (starting from four matrices representing genes and microbes from two independent datasets). The resulting edges per node are normally distributed, rather than power-law distributed (Supplementary Fig. S3). We calculated ranked microbes and genes according to degree centrality (correlations filtered to taxa associated with Fig. 2A are in Supplementary Table S2, the gene-microbe correlations with FDR-corrected *P* values are in Supplementary Table S3, and the full list of degree centrality results are in Supplementary Table S4). The microbe with the highest degree centrality, *Alistipes*, had edges to nearly 900 genes (Fig. 3C). *Alistipes* also was in the top 20% of all microbes by closeness centrality (Supplementary Table S5) and had the second-highest betweenness centrality (Supplementary Table S6). Other microbes with high degree centrality included species of *Bifidobacterium*, *Escherichia*, *Salmonella*, and *Fusobacterium*. For each of these top microbes, the pattern of relationships to genes in the network was distinct (Fig. 3C, colored lines). The genes connected to *Alistipes* represented apoptosis-related pathways, xenobiotic metabolism, and others (Fig. 3D; Supplementary Table S7). The genes linked to *Bifidobacterium* were distinct and related more strongly to inflammation (e.g., IFNγ response; Fig. 3D; Supplementary Table S7).

## Discussion

The {exotic} tool identifies low-abundance microbes within tumor biopsies using well-established alignment tools and several filtering steps. These include sample-based and microbe-based filtering, which utilizes fractions of microbial sequences in the tumors, the correlation with technical variables, and lists of common contaminants and common commensals to arrive at a dataset with a more than 50% reduction in observed microbes. Despite these efforts, we show large differences between analogous datasets from TCGA and ORIEN that may be due to contamination or other differences between the datasets, for example, the type of treatment patients received. Therefore, the analyses presented here rely on concordant results for both TCGA and ORIEN datasets concerning the effects’ direction and significance. With this framework, we explored the role of microbes in clinical outcomes and their association with clinical variables and immune cells. Furthermore, we built a pan-cancer microbe–gene interaction network to survey mechanisms by which microbes interact with tumors.

The methods used here are consistent with others that support the presence of microbes in many tumors. For example, Nejman and colleagues performed extensive amplicon- and IHC-based assessments of tumor samples, including nearly 1,000 controls. The result was detectable bacteria in over half of breast and bone cancers and fewer in melanoma and other cancers (8). On the other hand, computational-only methods that used one dataset reported microbes in all tested tumors (9). Others used multiple datasets in the contamination identification step, showing that microbial sequences in peripheral blood mononuclear cell samples from the same patients showed contaminant-like properties (20). As many datasets lack paired blood samples, we instead showed how the positive-associated based intersection method is an analogous method to reduce the FDR.

Notable differences with our strategy include focusing on RNA rather than DNA-based sequencing. This selection was a deliberate attempt to reduce contamination, as DNA may persist longer in the environment than RNA purification and sequencing apparatus (15, 24). However, both the ORIEN and TCGA datasets incorporated exon-enrichment in the sequencing library preparation, which would reduce the amounts of microbes. Therefore, it is important to consider these observations as a representation of the tumor microbiome, affected by technical features. Further, we included samples preserved by both FFPE and FF methods. The FFPE preservation method has been shown to contain more contaminants, according to 16S-based measurements (25). While the normalization methods appeared to control for large differences between FFPE and FF (Fig. 1C), the filtering strategies removed taxa flagged as likely contaminants across both preservation methods (Fig. 1B and D), which may have removed taxa that were only contaminants in one of the two types. The broadly lower prevalences of taxa observed in these datasets than by more targeted methods, such as quantitative PCR (Fig. 1E), may relate to the technical effects of exon selection, the inclusion of both FFPE and FF-preserved tissues, different rates of contamination in RNA-seq–based measurements relative to those described in the literature, or other reasons. The pipeline was constructed with modular filters, so less conservative filtering strategies could be applied to more uniformly collected datasets (e.g., all FF with sequenced negative controls).

In addition, we expanded the database of potential microbes to include fungi, owing to recent reports of fungi increasing tumor growth in pancreatic ductal adenocarcinoma (7) and affecting response to radiation in models of breast cancer and melanoma (26). Despite these differences, we found relatively well-characterized microbes in prevalences consistent with previous reports. Namely, we found *Fusobacterium* in roughly 30% and 60% of colorectal tumors for ORIEN and TCGA, respectively, but only 10% of non-colorectal tumors. This enrichment in colorectal tumors is consistent with other studies that used non–RNA-seq–based methods, such as quantitative PCR (16, 17), amplicon sequencing (18), or microscopy (19). The consistency with previous reports gave us the confidence to explore associations with clinical data.

Furthermore, the modular filtering strategy employed by {exotic} enables users to tailor the process to their datasets. While several steps would likely be useful in all contexts (e.g., including a “statistical filter” to exclude microbes whose abundance increases as the input RNA concentration is lower), others may be overly stringent. For example, the literature-based filter removes microbes commonly found in sequenced negative controls (15). Unfortunately, much of the sequencing was 16S-based, which lacks the resolution of RNA-seq–based approaches and is therefore overly stringent. Should users have access to negative controls for every technical batch; their sensitivity will likely be much greater. Theoretically, the {exotic} pipeline could be applied to non-RNA datasets, including whole genome shotgun sequencing. However, the default alignment parameters and filtering steps (e.g., statistical filtering with input RNA concentration) may not be optimal or applicable.

The presence or abundance of tumor microbes is associated with various clinical variables, including overall survival, age, BMI, and tumor features such as hypoxia and immune cell composition. While many microbes and taxonomic levels were associated with survival in the ORIEN and TCGA datasets separately, a much smaller number was observed in both, suggesting that caution should be observed with associations between tumor microbes inferred by this method and long-term survival in real-world datasets, particularly if only one dataset is available. Moreover, no microbes are consistently associated with survival in more than one cancer. This suggests microbe-cancer specificity that may provide a biomarker for cancer type or treatment outcomes. These observations are consistent with previous reports that showed machine learning models could predict cancer types with high accuracy (9). This association with survival does not take into account the treatment patients received. The two datasets were collected over different periods (2006–2013 for TCGA and 2003–2018 for ORIEN) when the dominant treatment shifted to immunotherapy in several cancers, including melanoma. Microbes have been shown to affect survival in the context of chemotherapy (27), radiation (26), and immunotherapy (2–4, 28, 29). Therefore, even tumor microbes consistently identified between the two datasets could show different results regarding overall survival.

Rank-based correlations with age and BMI also showed cancer specificity but in a different form than for survival. Many or few associations were found consistently between the two datasets for each cancer, depending on the clinical variable. In particular, age correlated with microbes across both datasets in BLCA and SKCM cancers but not in KIRC, LUAD, COAD, and SARC or all cancers. Older adults are more susceptible to urinary tract infections (30), while gut barrier function is maintained with healthy aging (31), consistent with significant correlations across some but not all cancers. Further investigation is needed to identify the association's cause within this subset of cancers.

Similarly, BMI is associated with BLCA, KIRC, LUAD, and SARC cancers, but not the others. A high BMI has been identified as a risk factor for several cancers, including COAD (32). Unlike any others described here, this analysis departs from the typical format of only showing agreement between the two datasets. BMI and many other clinical variables that have demonstrated importance in cancer are largely missing from TCGA clinical data. We, therefore, chose to call attention to a behavior similar to the age-based correlations using the ORIEN dataset alone and to emphasize the need for robust clinical information. Similar specificities were observed with the hypoxia and immune cell abundances, where microbes are associated with a fraction of cancers and immune cells. However, age was the most robust association between a clinical variable and tumor microbe abundances.

We sought to explore the mechanisms by which microbes and tumors interact using a network-based approach. By filtering to the strongest correlations, using them to build a network, and then using network features to identify the nodes with the most edges, we ranked microbes by their network importance. Several microbes with established interactions with tumors were among the nodes with a high degree centrality. For example, members of the genus *Bifidobacterium* correlated with genes in pathways related to IFN signaling and hypoxia. *Bifidobacterium* is a common gut commensal that was shown to affect the response to immune checkpoint blockade via the secretion of inosine, which is taken up by immune cells via the adenosine receptor A2AAR (33). In the tumor microenvironment, high levels of A2AAR ligands have been associated with hypoxia (34). The most connected microbe was *Alistipes*, a known gut commensal but without demonstrated effects in tumors. *Alistipes* were linked to genes involved in many fundamental processes in the cell, including metabolism. Further research is needed to verify whether this bacterial genus demonstrates these effects within tumors.

This work is limited in several ways. First, there is a high potential for false positives. Our attempts to mitigate this risk included starting with RNA-based sequencing, the pipeline design with stringent alignment thresholds and conservative filters, validation by literature review and 16S sequencing, and requiring concordance between two independent datasets. However, all the analyses would benefit from experimental validation of the presence of microbes or their association. Second, some analyses, such as the association with overall survival, rely on assumptions about the presence of an organism being important rather than some measure of its quantity or location, which has recently been shown to affect outcomes (35). Third, not all analyses could be confirmed across datasets because of missing clinical values. This includes treatment information, and there are many examples of microbes affecting outcomes conditional on some treatment regimen (e.g., γ-*Proteobacteria* affecting response to treatment with gemcitabine; ref. 27). Finally, many tumor samples are collected years before the patient dies; the turnover rate for most intrinsic tumor microbes has not been established, so the microbes observed here may not be present for long. These limitations affected some analyses more than others. The associations with overall survival were sensitive to nearly every limitation, which may explain the relatively small number of concordant associations observed in both the TCGA and ORIEN datasets.

The {exotic} tool identifies microbes in the context of human-dominated high-throughput sequencing datasets, such as tumor RNA-seq. We further solidified the consistent presence of microbes in tumor samples by processing and intersecting analyses in two large, independent datasets. In addition, we observed new correlations with age, BMI, and features of the tumor microenvironment, such as hypoxia and immune cell abundance. The microbes may interact with hundreds of genes, affecting pathways such as metabolism and hypoxia. This is another example of the value of large, publicly available data repositories such as TCGA and how they can yield new information many years after they are generated. In the case of {exotic}, several new lines of evidence allow the reanalysis of these data, including (i) more complete microbial genome databases, (ii) thorough contaminant profiling datasets, and (iii) smaller-scale validation studies that increase the confidence in low-abundance organisms. Still, repurposing tumor sequencing data to observe non-human sequences has many challenges. Major barriers to understanding when and how tumor microbes affect cancer are the (i) sparsity of the data and (ii) heterogeneity at the level of patient demographics, tumors, and treatments. In addition, the source of many microbes, whether they originate from the gut, oral cavity, or elsewhere, remains an open question. Geography has been shown to affect the gut and other microbiomes and is expected to drive differences in the tumor microbiome (36). We propose that many challenges can be overcome with sufficiently large, well-annotated, and diverse tumor sequencing datasets. Additional work is needed to validate these observations and identify the mechanisms by which they may affect cancer outcomes.

## Supplementary Material

Supplemental Figure S1 Example survival analysis using multiple stratification thresholds. The p-values for the separations between survival curves at each quantile are shown, colored by the direction of association (hazard ratio greater or less than 1).

Supplemental Figure S2 Histogram of the correlations between microbes and genes, from which the most extreme 5% were selected for the network.

Supplemental Figure S3 The number of edges per node for the nodes of a random network.

Supplementary Table S1 Individual results for the associations of microbe prevalence with survival (on GitHub as spakowiczlab/exotic-manuscript/analyses/data).

Supplementary Table S2 Summary of the microbes associated with survival and the concordance network-based rankings.

Supplementary Table S3 Ranked list of microbes by degree centrality in a gene-microbe network controlling for cancer type (on GitHub as spakowiczlab/exotic-manuscript/analyses/tables/S3_network_microbe_degree-centrality.csv).

Supplementary Table S4 Ranked list of microbes by closeness centrality in a gene-microbe network controlling for cancer type (on GitHub as spakowiczlab/exotic-manuscript/analyses/tables/S4_network_microbe_closeness-centrality.csv)

Supplementary Table S5 Ranked list of microbes by betweenness centrality in a gene-microbe network controlling for cancer type (on GitHub as spakowiczlab/exotic-manuscript/analyses/tables/S4_network_microbe_betweeenness-centrality.csv)

Supplementary Table S6 Pathways enriched in the genes linked to microbes with the highest degree centrality.

Supplementary Table S7 Gene set enrichment analysis of the MSigDB Hallmark pathways using genes significantly associated with microbes in the TCGA and ORIEN datasets.
