# Supplementary figures and images for: Exogenous Sequences in Tumors and Immune Cells (Exotic): A Tool for Estimating the Microbe Abundances in Tumor RNA-seq Data
Source: Cancer Res Commun. 2023 Nov 21;3(11):2375–85. doi: 10.1158/2767-9764.CRC-22-0435 (PMC10662017; doi:10.1158/2767-9764.CRC-22-0435)

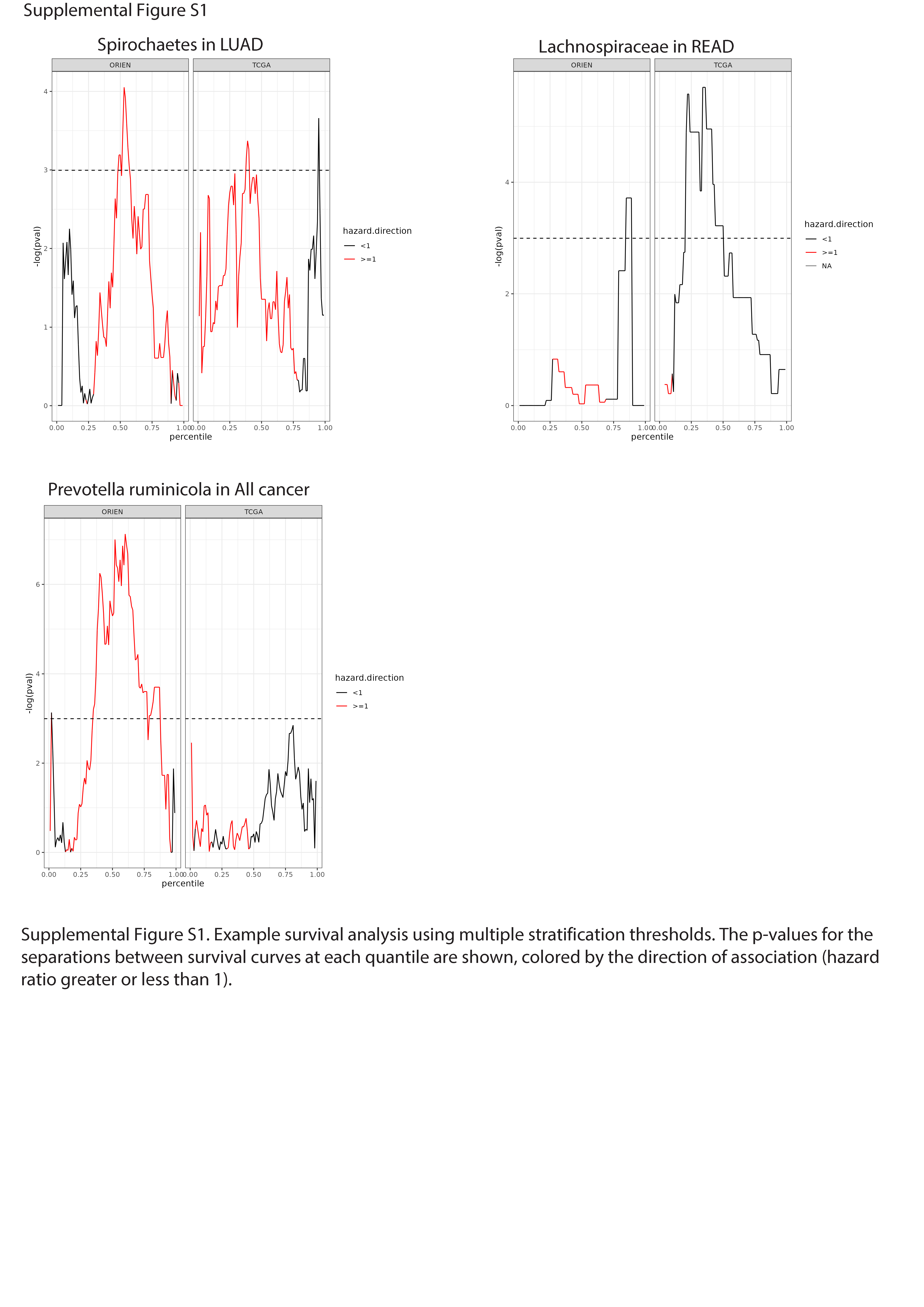

Supplement: Supplemental Figure S1 — Example survival analysis using multiple stratification thresholds. The p-values for the separations between survival curves at each quantile are shown, colored by the direction of association (hazard ratio greater or less than 1). [file crc-22-0435-s01.png]

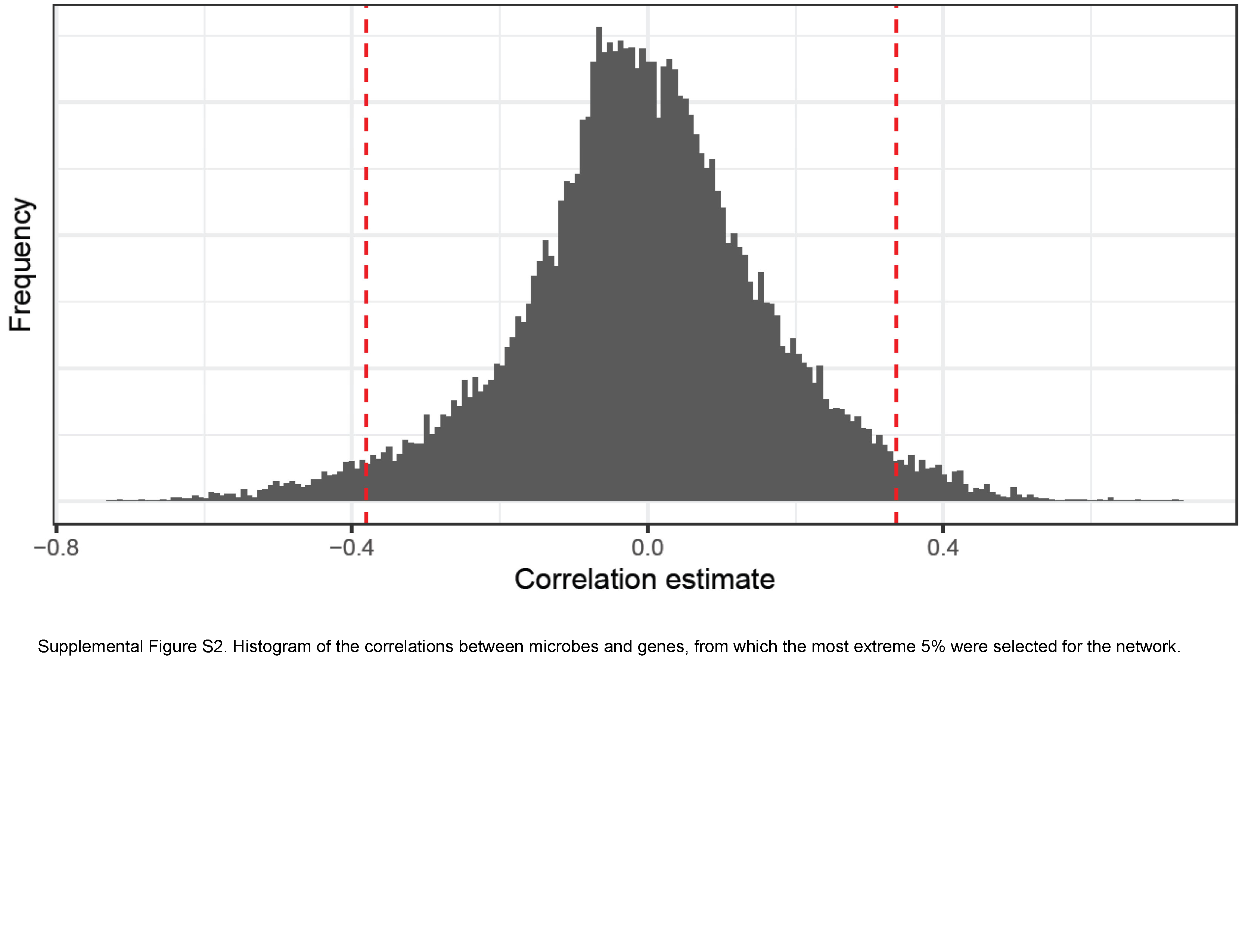

Supplement: Supplemental Figure S2 — Histogram of the correlations between microbes and genes, from which the most extreme 5% were selected for the network. [file crc-22-0435-s02.png]

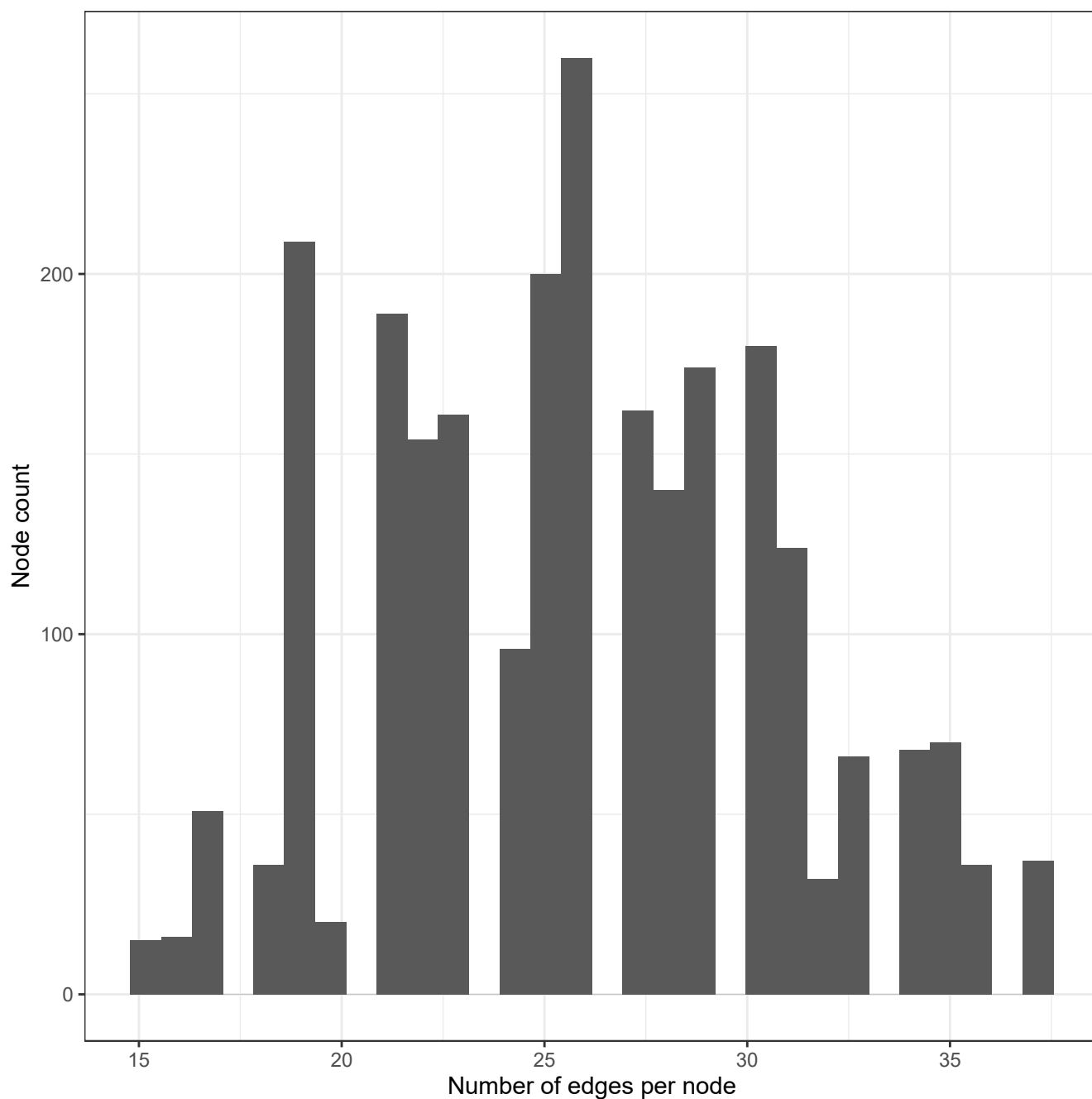

Supplemental figure S3. The number of edges per node for the nodes of a random network.

Supplement: Supplemental Figure S3 — The number of edges per node for the nodes of a random network. [file crc-22-0435-s03.pdf]
